# Ultra-high sensitive cancerous cells detection and sensing capabilities of photonic biosensor

**DOI:** 10.1038/s41598-023-46667-y

**Published:** 2023-11-09

**Authors:** Arafa H. Aly, B. A. Mohamed, M. Al-Dossari, S. K. Awasthi, Emadelden Fouad, A. F. Amin

**Affiliations:** 1 TH-PPM Group, Physics Department, Faculty of Sciences, Beni-Suef University, Beni Suef, 62514 Egypt; 2 Department of Physics, Faculty of Science, King Khalid University, 62529 Abha, Saudi Arabia; 3 Department of Physics and Material Science and Engineering, Jaypee Institute of Information Technology, Noida, 201304 India; 4 Department of Engineering Physics, Florida Polytechnic University, Lakeland, USA; 5 Faculty of Technology and Education, Beni-Suef University, Beni Suef, 62521 Egypt

**Keywords:** Materials science, Optics and photonics, Physics

## Abstract

The ultra-high sensitive cancer cell detection capabilities of one-dimensional photonic crystal with defect have been theoretically examined in this work. The simulations of the work have been carried out with MATLAB programming and transfer matrix method. The performance of the proposed biosensor loaded separately with samples containing different cancer cells has been studied by changing the period number, defect layer thickness, and incident angle corresponding to s polarized light only to identify the parameters under which the proposed design becomes ultra-sensitive. The working principle of the proposed biosensor is to sense the minute change in the refractive index of the analytes containing different cancer cells of human. This sensing is done shifting the respective defect mode inside photonic band gap of the structure from one position to other near by position due to change in the refractive index of sample under consideration. Our structure under optimum conditions yields maximum shifting in the position of defect mode from 1538 to 1648 nm corresponding to the samples containing normal and Glioblastoma cells of refractive indices 1.350 and 1.4470 respectively which results a ultra-high sensitivity of 4270.525928 nm/RIU.

## Introduction

The study of controlled and rapid propagation of information via light is one the most emerging areas of research and development in the field of photonic device technology^1^. The current developments in the material science and engineering have attracted many researchers working in the photonic engineering to develop optoelectronic devices of nano-scale dimensions^2^. Photonic crystals (PCs) have the magnificent capabilities of handling the time orientated technological challenges successfully as per the industrial demand. The ease of fabrication involved in one-dimensional (1d) photonic structures has given them edge over two-dimensional (2d) and three-dimensional (3d) photonic structures^3^. The participation of different materials layers in the unit cell of the PC may allow them to group into binary, ternary, quaternary photonic structures. The directional repetition of unit cell into large number is responsible for formation of 1d, 2d or 3d photonic structures. PCs have an especial property of photonic band gaps (PBGs) due to the periodic repetition of layers of different refractive indices leading to the constructive and destructive interference between the rays of electromagnetic waves (EMWs) refracted and reflected from the interfaces separating any two media of the structure respectively because of multiple Bragg scattering^4,5^. The frequencies of EMWs interfere destructively are not allowed to pass through the structure and are responsible for creation of PBG. If the deformity is created into the photonic structure, a defect mode of high transmittance appears inside PBG. The position and intensity of the defect mode is dependent upon the refractive index of the deformity^6–9^. This excellent property of PCs has attracted the attention of the researchers to use them for various biosensing applications^10,11^.

Cancer is a most common diseases which allows the uncontrollable growth of cell in human body and has ability to infect any part of the human body^12,13^. Actually, human body allow the growth of new cell which multiply together to form new cells as per the requirement of the body. The lifetime of every cell is fixed so when the cells become old or damaged body may develop new cells automatically to replace older one^14^. But the present life style is breaking down this natural process of cell regeneration and allowing the abnormal growth of damaged or deadly cells in the human body^15^. The lumps of trillions of damaged cells or tissues inside human body are called as tumor which may be cancerous or non-cancerous^16^. Cancerous tumor is also called as malignant which has life threatening impact on human life. Most of the cancers are in solid form, malignancy may also exist is human in medical science blood cancer disease is known as leukemia^17^. Human body send signals to the normal cells for their development, cancerous cells start developing without getting any signal of their development. Normally deadly cells are extracted or eliminated by our immune system, extraction is not done in the case of cancerous cells^18^. The prevention of malignancy can be only achieved if we could identify the growth of deadly cells in time. The early stage detection of malignancy is only possible if we could have full proof diagnosis mechanism which could provide precise, rapid and cost-effective results. Presently several cancer cell detection techniques are being used in the early stage and timely detection of cancerous cells in human body. Nowadays, microfluidic, plasmonic and photonic biosensing, immunocyte-chemistry and electrochemical technique-based devices are being used for early and timely detection of cancers cells in human body^19–22^. Recently Chung-Ting et al. presented a sensitive plasmonic biosensing design whose unit cell is composed of closed loop dual band perfect absorber using intersection nanostructure for detection of malignant cells of human body^23^. Moreover, Malek et al. have discussed the high sensitive biosensor composed of 1d defective ternary photonic crystal for early stage detection of cancerous cells of sensitivity 3282.09 nm/RIU^24^.

The work of Malek et al. motivated us for this study to design ultra-sensitive biosensor for cancer cell detection. In the current study, the optical properties of the 1d defective ternary photonic crystal (DTPC) have been used for the detection of cancerous cell in human body^24^. We have used in the calculations and simulations the transfer matrix formulation in addition to MATLAB software. Both normal and oblique incidence corresponding to s polarized incident EMWs have been considered in this study. For achieving ultra-high sensitivity from the 1d DTPC while investigating various cancerous cells we have studied the effect of change in period number, incident angle and the thickness of the defect layer on the performance of the design. The organization of the paper is as under. “Introduction” section deals with introduction. Structural design and theoretical formulation are presented in “Structural design and its realization” and “Theoretical formulation” sections respectively. Results and discussions are written in “Results and discussions” section. Finally, conclusions are discussed in “Conclusion” section of this manuscript.

## Structural design and its realization

**Figure 1:**
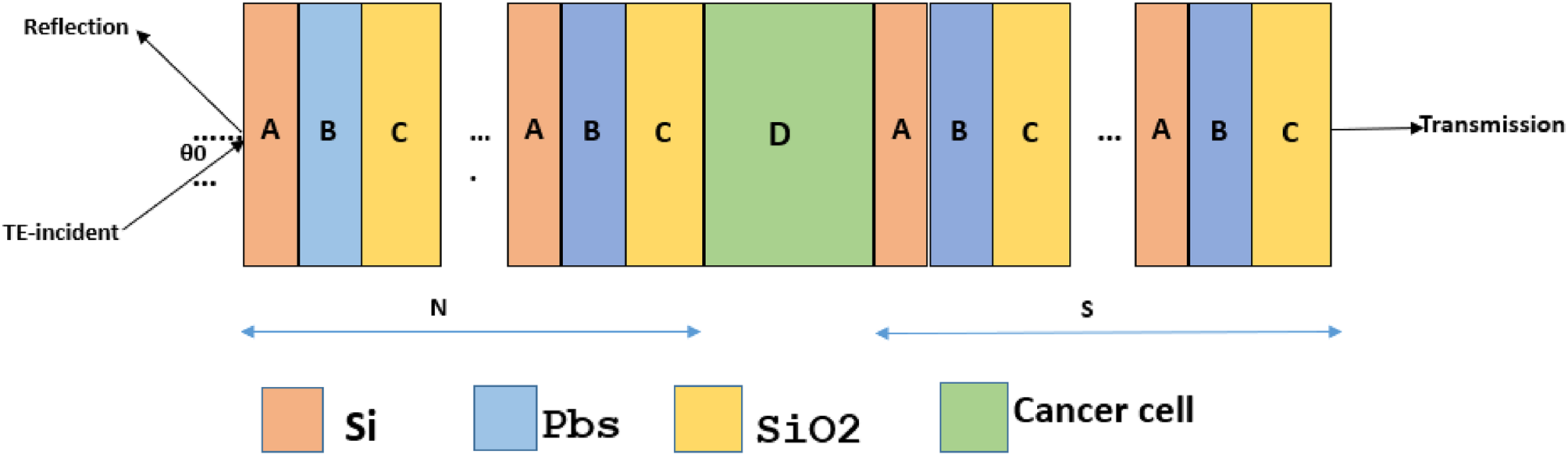
Schematic diagram of a 1d defective ternary photonic crystal with defect. The colours orange, blue, yellow and green are used to show A, B, C and D layers of the structure representing silicon, lead sulphide, silicon di-oxide and defect layer.

The proposed design has been configured by introducing a defect layer D of air at the centre of two ternary photonic crystals such that (ABC)^*N*^/D/(ABC)^*S*^/Substrate. Here alphabets A, B, and C are being used to represent layers of materials Si, Pbs and SiO_2_ respectively. The letters *N* and *S* are used to represent period number which is equal in our case. The architecture of the proposed design air/(Si/Pbs/SiO_2_)^*N*^/Defect/(Si/Pbs/SiO_2_)^S^/air is shown in Fig. 1. The refractive indices and thicknesses of layers A, B, C and D of are denoted by *n*_1_, *n*_2_, *n*_3,_ *n*_d_ and *d*_1_, *d*_2_, *d*_3_, *d*_d_ respectively. The refractive indices of the ambient media and substrate of the structure are denoted by *n*_0_ and *n*_s_ respectively which are air in this case. The numeric values of refractive indices and thicknesses of different layers including semi-infinite media are being presented in Table 1 below.

**Table 1:** Description of refractive indices and thicknesses of different material layers.

Notation	Material	Reflective indices	Thicknesses (nm)	References
Ambient	Air	*n*_0_ = 1	–	
Substrate	Air	*n*_s_ = 1	–	
*A*	Si	*n*_1_ = 3.3	*d*_1_ = 95	^25^
*B*	PbS	*n*_2_ = 4.2	*d*_2_ = 155	^6^
*C*	SiO_2_	*n*_3_ = 1.46	*d*_3_ = 235	^26^
*D*	Analyte	*n*_d_ = 1.350	*d*_D_ = 1D = 800 nm	^27^

## Theoretical formulation

For the simulation of results pertaining to the proposed research work based on TMM, we have used MATLAB programming by using transfer matrix method. The electric and magnetic fields amplitudes at incident media to exit media are connected of through resulting transfer matrix *F* as^6,25–27^

1 $$F=\left(\begin{array}{c}{F}_{11}{F}_{12}\\ {F}_{21}{F}_{22}\end{array}\right)={\left({f}_{1}{f}_{2}{f}_{3}\right)}^{N}\left({f}_{D}\right){\left({f}_{1}{f}_{2}{f}_{3}\right)}^{S}.$$

Here *F*_11_, *F*_12_, *F*_21_ and *F*_22_ are the elements of matrix *F* representing whole structure. The 2 by 2 matrix representing individual layers *A*, *B*, *C* and *D* of DTPCs have been shown by *f*_1_, *f*_2_, *f*_3_ and *f*_D_ respectively. The transmission coefficient representing proposed 1D DTPC air/(Si/Pbs/SiO_2_)^*N*^/Defect/(Si/Pbs/SiO_2_)^S^/air can be evaluated with the help of following expression as^28–30^

2 $$t=\frac{2{\alpha }_{I}}{\left({F}_{11}+{F}_{12}{\alpha }_{S}\right){\alpha }_{I}+\left({F}_{21}+{F}_{22}{\alpha }_{S}\right)},$$

where admittance of incident and exit ends of the structure corresponding to s polarized ware are shown by ${\alpha }_{I}={n}_{0}{\text{cos}}{\gamma }_{0}$ and ${\alpha }_{S}={n}_{S}{\text{cos}}{\gamma }_{S}$ respectively and for p polarized incident wave, ${\alpha }_{I}=\frac{{\text{cos}}{\gamma }_{0}}{{n}_{0}}$ and ${\alpha }_{I}=\frac{{\text{cos}}{\gamma }_{S}}{{n}_{S}}$. The symbols γ_0_ and γ_S_ are representing angle of incident and angle of emergence respectively.

Finally, transmittance can be obtained as^30–32^

3 $$T=\left(\frac{{\alpha }_{S}}{{\alpha }_{I}}t{t}^{*}\right)\times 100.$$

The performance of the biosensor based on 1D TPC has been evaluated with the help of one of the most popular parameters named as sensitivity. Actually, while observing the sensitivity of any biosensing structure the ratio of change in the central wavelength of resonant peak ($\delta \lambda$) due to alteration in the refractive index of the sample ($\delta n$) under investigation is named as sensitivity of the design. It determines how minutely the biosensor can detect the change in the refractive index of the sample under investigation. It is defined as^33–35^

4 $$S=\frac{\delta \lambda }{\delta n}\left(\text{nm/RIU}\right).$$

## Results and discussions

The refractive indices of different samples containing various cancerous cells of human body examined in this work are presented in Table 2.

**Table 2:** Refractive index details of samples containing various cells^24^.

Description of sample	Refractive index
Normal	1.350
Jurkat	1.390
Hela	1.392
PC12	1.395
MDA-MB231	1.399
MCF-7	1.401
White matter	1.4121
Low grade glioma	1.4320
Glioblastoma	1.4470

**Figure 2:**
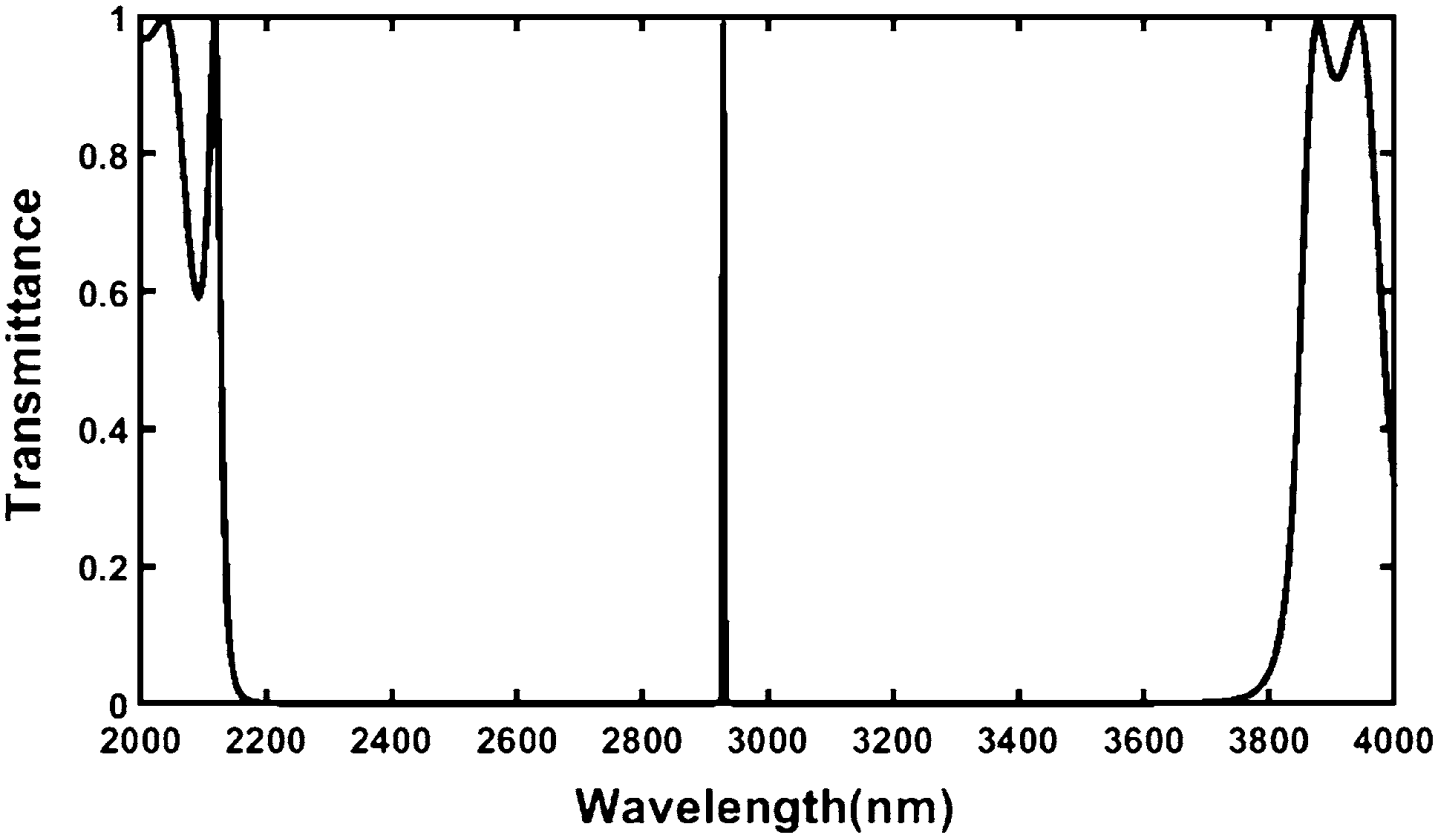
Transmission spectrum of the proposed design {air/(Si/Pbs/SiO_2_)^*N*^/Defect /(Si/Pbs/SiO_2_)^S^/air} loaded with sample containing normal cell under normal incidence (θ_0_ = 0°, d_1_ = 95 nm, d_2_ = 155 nm, d_3_ = 235 nm, d_*D*_ = 800 nm, n_1_ = 3.3, n_2_ = 4.2, n_3_ = 1.46 and N = S = 5).

In this paper we have examined the change in the position of defect mode due to the corresponding change in the sample poured into cavity region *D* of the structure. First, we have loaded the proposed design with a sample containing normal cell of human body and simulated the transmission spectra showing a defect mode of unit transmittance positioned at a wavelength 2928.7386 nm inside PBG of the structure extending from 2155 to 3800 nm as shown in Fig. 2.

**Figure 3:**
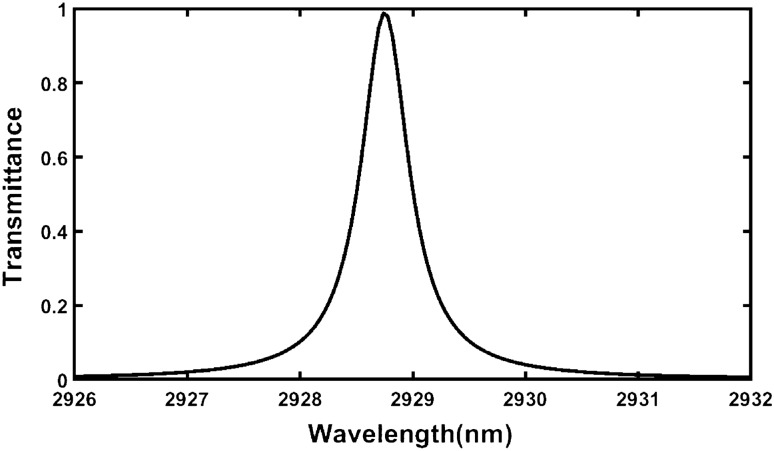
An enlarged view of Fig. 2 showing the defect mode position inside PBG of proposed design {air/(Si/Pbs/SiO_2_)^*N*^/Defect /(Si/Pbs/SiO_2_)^S^/air} at *θ*_0_ = 0°.

An enlarged view of Fig. 2 has been shown in Fig. 3 below. It is showing the clear picture of defect mode centered at 2928.7386 nm. This figure is very useful for obtaining the numeric values of central wavelength of the defect mode and its full width half maximum as mentioned above.

**Figure 4:**
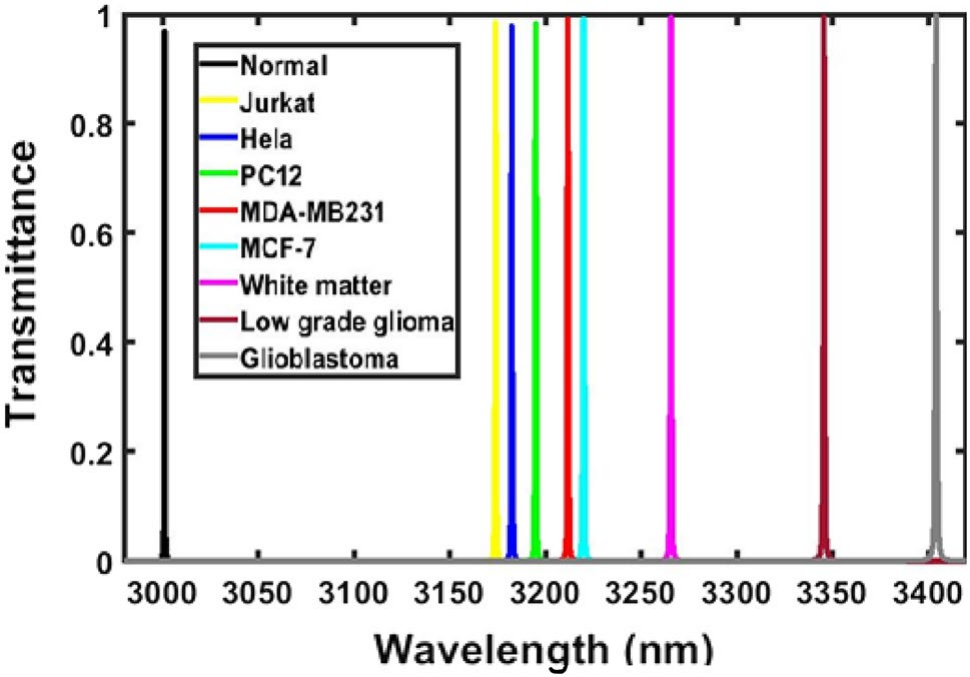
Transmission spectra of the proposed design {air/(Si/Pbs/SiO_2_)^*N*^/Defect /(Si/Pbs/SiO_2_)^S^/air} loaded with samples containing normal, Jurkat, Hela, PC12, MDA-MB231, MCF-7, White matter, Low grade glioma and Glioblastoma cells independently under normal incidence (*θ*_0_ = 0°, *d*_1_ = 95 nm, d_2_ = 155 nm, d_3_ = 235 nm, *d*_D_ = 800 nm, n_1_ = 3.3, n_2_ = 4.2, n_3_ = 1.46 and *N* = 5).

Next, we have examined the response of the proposed design loaded with samples containing normal, Jurkat, Hela, PC12, MDH-MB231, MCF-7, White matter, Low grade glioma, Glioblastoma cells of human body separately with respect to sample containing normal cells of human body under normal incidence condition. The transmittance response of the proposed biosensor loaded separately with the samples as per the details given in Table 2 is shown in Fig. 4. It has been observed from Fig. 4 that as sample containing cancerous cells Jurkat to Glioblastoma the respective defect modes start shifting towards higher wavelength side inside PBG of the structure. This movement of defect mode corresponding to samples of different cells is governed by the condition of standing wave inside laser cavity as discussed in Eq. (5) below^28–35^

5 $$\varphi =s\lambda ={n}_{eff}\rho .$$

Here symbols φ and ρ are being used to show optical and geometrical path differences between the propagating waves inside structure. The notations *s*, *λ* and *n*_eff_ are representing free space wavelength of the light injected into the structure, an integer and effective refractive index of the structure respectively. The increase in the refractive index of the sample poured into the defect layer region *D* of the structure also increases the *n*_eff_ which in turn allow the movement of the defect mode inside PBG towards higher wavelength side to keep φ remains same. The movement of all defect modes dependent upon the sample poured into the cavity region *D* of the structure is measured with respect to the defect mode arises due to sample containing normal cell as shown with black colour in Fig. 4.

The defect mode position shift in the central wavelength of the defect mode position due to change in the refractive index of the sample with respect to the central wavelength of defect mode associated with the sample containing normal cell and the sensitivity of the structure loaded with various samples independently are being summarized in Table 3 below. All these values have been obtained with the help of Fig. 4 in addition to Eq. (5).

**Table 3:** Performance evaluation table showing position of defect mode, shift in the position of defect mode due to change in the refractive index of sample containing various cancerous cells with respect to the sample containing normal cell and the sensitivity of the proposed structure loaded with different cells under normal incidence *θ*_0_ = 0°.

Cell	Refractive index	Defect mode positions (nm)	Wavelength shift (nm)	Sensitivity (nm/RIU)
Normal	1.350	2928.7386	–	–
Jurkat	1.390	2975.9795	47.2409	1181.0225
Hela	1.392	2978.3396	49.601	1180.97619
PC12	1.395	2981.8196	53.081	1179.577778
MDA-MB231	1.399	2986.4997	57.7611	1178.797959
MCF-7	1.401	2988.8198	60.0812	1178.062745
White matter	1.4121	3001.7	72.9614	1174.901771
Low grade glioma	1.4320	3024.5805	95.8419	1168.803659
Glioblastoma	1.4470	3041.6608	112.9222	1164.146392

It is evident from the data of Table 3 that the sensitivity of proposed structure loaded separately with different samples varies between maximum of 1181.0225 and 1164.146392 nm/RIU corresponding to the samples containing Jurkat to Glioblastoma cells respectively under normal incidence.

### The effect of the incident angle on the sensitivity

Next, attempts have been further given to improve the sensitivity of the proposed structure loaded independently with samples containing various cancerous cells with respect to normal cell. This purpose has been achieved by increasing the angle of incidence from *θ*_0_ = 0° to *θ*_0_ = 85° in steps of 5° corresponding to s-polarized light only. The defect mode position representing the shift in the central wavelength of the defect mode due to change in the refractive index of the samples with respect to the central wavelength of defect mode associated with the sample containing normal cell and sensitivity of the proposed structure loaded with samples as discussed in Table 2 corresponding to incident angle *θ*_0_ = 5°, 10°, 15°, 20°, 25°, 30°, 35°, 40°, 45°, 50°, 55°, 60°, 65°, 70°, 75°, 80° and 85° under s-polarized wave are given in Tables 4, 5, 6, 7, 8, 9, 10, 11, 12, 13, 14, 15, 16, 17, 18, 19 and 20 respectively. It has been observed from Tables 4, 5, 6, 7, 8, 9, 10, 11, 12, 13, 14, 15, 16, 17, 18, 19 and 20 that as incident angle increases from 0° to 85° the performance of the structure also increases as the sensitivity increases. The sensitivity reaches to maximum of 1838.387629 nm/RIU when the cavity is loaded with the sample containing Glioblastoma cell as evident from Table 20.

**Table 4:** Performance evaluation table showing position of defect mode, shift in the position of defect mode due to change in the refractive index of sample containing various cancerous cells with respect to the sample containing normal cell and the sensitivity of the proposed structure loaded with different cells under normal incidence *θ*_0_ = 5°.

Cell	Refractive index	Defect mode positions (nm)	Wavelength shift (nm)	Sensitivity (nm/RIU)
Normal	1.35	2924.7385	–	–
Jurkat	1.39	2972.1394	47.4009	1185.0225
Hela	1.392	2974.4995	49.761	1184.785714
PC12	1.395	2978.0196	53.2811	1184.024444
MDA-MB231	1.399	2982.6997	57.9612	1182.881633
MCF-7	1.401	2985.0197	60.2812	1181.984314
White matter	1.4121	2997.94	73.2015	1178.768116
Low grade glioma	1.432	3020.9004	96.1619	1172.706098
Glioblastoma	1.447	3038.0208	113.2823	1167.858763

**Table 5:** Performance evaluation table showing position of defect mode, shift in the position of defect mode due to change in the refractive index of sample containing various cancerous cells with respect to the sample containing normal cell and the sensitivity of the proposed structure loaded with different cells under normal incidence *θ*_0_ = 10°.

Cell	Refractive index	Defect mode positions (nm)	Wavelength shift (nm)	Sensitivity (nm/RIU)
Normal	1.35	2912.6983	–	–
Jurkat	1.39	2960.6192	47.9209	1198.0225
Hela	1.392	2963.0193	50.321	1198.119048
PC12	1.395	2966.5793	53.881	1197.355556
MDA-MB231	1.399	2971.2994	58.6011	1195.940816
MCF-7	1.401	2973.6595	60.9612	1195.317647
White matter	1.4121	2986.6997	74.0014	1191.648953
Low grade glioma	1.432	3009.9402	97.2419	1185.876829
Glioblastoma	1.447	3027.2605	114.5622	1181.053608

**Table 6:** Performance evaluation table showing position of defect mode, shift in the position of defect mode due to change in the refractive index of sample containing various cancerous cells with respect to the sample containing normal cell and the sensitivity of the proposed structure loaded with different cells under normal incidence *θ*_0_ = 15°.

(C) Cell	Refractive index	Defect mode positions (nm)	Wavelength shift (nm)	Sensitivity (nm/RIU)
Normal	1.35	2892.8179	–	–
Jurkat	1.39	2941.6188	48.8009	1220.0225
Hela	1.392	2944.0189	51.201	1219.071429
PC12	1.395	2947.619	54.8011	1217.802222
MDA-MB231	1.399	2952.459	59.6411	1217.165306
MCF-7	1.401	2954.8591	62.0412	1216.494118
White matter	1.4121	2968.1394	75.3215	1212.906602
Low grade glioma	1.432	2991.7798	98.9619	1206.852439
Glioblastoma	1.447	3009.4202	116.6023	1202.085567

**Table 7:** Performance evaluation table showing position of defect mode, shift in the position of defect mode due to change in the refractive index of sample containing various cancerous cells with respect to the sample containing normal cell and the sensitivity of the proposed structure loaded with different cells under normal incidence *θ*_0_ = 20°.

Cell	Refractive index	Defect mode positions (nm)	Wavelength shift (nm)	Sensitivity (nm/RIU)
Normal	1.35	2865.2973	–	–
Jurkat	1.39	2915.2583	49.961	1249.025
Hela	1.392	2917.7384	52.4411	1248.597619
PC12	1.395	2921.4584	56.1611	1248.024444
MDA-MB231	1.399	2926.3785	61.0812	1246.555102
MCF-7	1.401	2928.8586	63.5613	1246.3
White matter	1.4121	2942.4988	77.2015	1243.180354
Low grade glioma	1.432	2966.6993	101.402	1236.609756
Glioblastoma	1.447	2984.7797	119.4824	1231.77732

**Table 8:** Performance evaluation table showing position of defect mode, shift in the position of defect mode due to change in the refractive index of sample containing various cancerous cells with respect to the sample containing normal cell and the sensitivity of the proposed structure loaded with different cells under normal incidence *θ*_0_ = 25°.

Cell	Refractive index	Defect mode positions (nm)	Wavelength shift (nm)	Sensitivity (nm/RIU)
Normal	1.35	2830.4966	–	–
Jurkat	1.39	2882.0176	51.521	1288.025
Hela	1.392	2884.5777	54.0811	1287.645238
PC12	1.395	2888.3778	57.8812	1286.248889
MDA-MB231	1.399	2893.4579	62.9613	1284.92449
MCF-7	1.401	2896.0179	65.5213	1284.731373
White matter	1.4121	2910.0582	79.5616	1281.185185
Low grade glioma	1.432	2935.0187	104.5221	1274.659756
Glioblastoma	1.447	2953.6591	123.1625	1269.716495

**Table 9:** Performance evaluation table showing position of defect mode, shift in the position of defect mode due to change in the refractive index of sample containing various cancerous cells with respect to the sample containing normal cell and the sensitivity of the proposed structure loaded with different cells under normal incidence *θ*_0_ = 30°.

Cell	Refractive index	Defect mode positions (nm)	Wavelength shift (nm)	Sensitivity (nm/RIU)
Normal	1.35	2789.0158	–	–
Jurkat	1.39	2842.3368	53.321	1333.025
Hela	1.392	2844.9769	55.9611	1332.407143
PC12	1.395	2848.937	59.9212	1331.582222
MDA-MB231	1.399	2854.1771	65.1613	1329.822449
MCF-7	1.401	2856.8171	67.8013	1329.437255
White matter	1.4121	2871.3774	82.3616	1326.273752
Low grade glioma	1.432	2897.2179	108.2021	1319.537805
Glioblastoma	1.447	2916.5383	127.5225	1314.664948

**Table 10:** Performance evaluation table showing position of defect mode, shift in the position of defect mode due to change in the refractive index of sample containing various cancerous cells with respect to the sample containing normal cell and the sensitivity of the proposed structure loaded with different cells under normal incidence *θ*_0_ = 35°.

Cell	Refractive index	Defect mode positions (nm)	Wavelength shift (nm)	Sensitivity (nm/RIU)
Normal	1.35	2741.5348	–	–
Jurkat	1.39	2796.8959	55.3611	1384.0275
Hela	1.392	2799.656	58.1212	1383.838095
PC12	1.395	2803.7361	62.2013	1382.251111
MDA-MB231	1.399	2809.2162	67.6814	1381.253061
MCF-7	1.401	2811.9762	70.4414	1381.203922
White matter	1.4121	2827.0965	85.5617	1377.805153
Low grade glioma	1.432	2853.9771	112.4423	1371.247561
Glioblastoma	1.447	2874.0575	132.5227	1366.213402

**Table 11:** Performance evaluation table showing position of defect mode, shift in the position of defect mode due to change in the refractive index of sample containing various cancerous cells with respect to the sample containing normal cell and the sensitivity of the proposed structure loaded with different cells under normal incidence *θ*_0_ = 40°.

Cell	Refractive index	Defect mode positions (nm)	Wavelength shift (nm)	Sensitivity (nm/RIU)
Normal	1.35	2688.9738	–	–
Jurkat	1.39	2746.6549	57.6811	1442.0275
Hela	1.392	2749.495	60.5212	1440.980952
PC12	1.395	2753.7751	64.8013	1440.028889
MDA-MB231	1.399	2759.4952	70.5214	1439.212245
MCF-7	1.401	2762.3352	73.3614	1438.458824
White matter	1.4121	2778.0956	89.1218	1435.133655
Low grade glioma	1.432	2806.1361	117.1623	1428.808537
Glioblastoma	1.447	2827.0965	138.1227	1423.945361

**Table 12:** Performance evaluation table showing position of defect mode, shift in the position of defect mode due to change in the refractive index of sample containing various cancerous cells with respect to the sample containing normal cell and the sensitivity of the proposed structure loaded with different cells under normal incidence *θ*_0_ = 45°.

Cell	Refractive index	Defect mode positions (nm)	Wavelength shift (nm)	Sensitivity (nm/RIU)
Normal	1.35	2632.6527	–	–
Jurkat	1.39	2692.7339	60.0812	1502.03
Hela	1.392	2695.7339	63.0812	1501.933333
PC12	1.395	2700.174	67.5213	1500.473333
MDA-MB231	1.399	2706.1341	73.4814	1499.620408
MCF-7	1.401	2709.0942	76.4415	1498.852941
White matter	1.4121	2725.5745	92.9218	1496.325282
Low grade glioma	1.432	2754.8551	122.2024	1490.273171
Glioblastoma	1.447	2776.6955	144.0428	1484.97732

**Table 13:** Performance evaluation table showing position of defect mode, shift in the position of defect mode due to change in the refractive index of sample containing various cancerous cells with respect to the sample containing normal cell and the sensitivity of the proposed structure loaded with different cells under normal incidence *θ*_0_ = 50°.

Cell	Refractive index	Defect mode positions (nm)	Wavelength shift (nm)	Sensitivity (nm/RIU)
Normal	1.35	2574.0115	–	–
Jurkat	1.39	2636.6127	62.6012	1565.03
Hela	1.392	2639.7328	65.7213	1564.792857
PC12	1.395	2644.3729	70.3614	1563.586667
MDA-MB231	1.399	2650.573	76.5615	1562.479592
MCF-7	1.401	2653.6931	79.6816	1562.384314
White matter	1.4121	2670.8534	96.8419	1559.450886
Low grade glioma	1.432	2701.414	127.4025	1553.689024
Glioblastoma	1.447	2724.2545	150.243	1548.896907

**Table 14:** Performance evaluation table showing position of defect mode, shift in the position of defect mode due to change in the refractive index of sample containing various cancerous cells with respect to the sample containing normal cell and the sensitivity of the proposed structure loaded with different cells under normal incidence *θ*_0_ = 55°.

Cell	Refractive index	Defect mode positions (nm)	Wavelength shift (nm)	Sensitivity (nm/RIU)
Normal	1.35	2514.9703	–	–
Jurkat	1.39	2579.9716	65.0013	1625.0325
Hela	1.392	2583.2117	68.2414	1624.795238
PC12	1.395	2588.0518	73.0815	1624.033333
MDA-MB231	1.399	2594.5319	79.5616	1623.706122
MCF-7	1.401	2597.732	82.7617	1622.778431
White matter	1.4121	2615.6123	100.642	1620.644122
Low grade glioma	1.432	2647.4529	132.4826	1615.641463
Glioblastoma	1.447	2671.2534	156.2831	1611.165979

**Table 15:** Performance evaluation table showing position of defect mode, shift in the position of defect mode due to change in the refractive index of sample containing various cancerous cells with respect to the sample containing normal cell and the sensitivity of the proposed structure loaded with different cells under normal incidence *θ*_0_ = 60°.

Cell	Refractive index	Defect mode positions (nm)	Wavelength shift (nm)	Sensitivity (nm/RIU)
Normal	1.35	2457.5692	–	–
Jurkat	1.39	2524.8105	67.2413	1681.0325
Hela	1.392	2528.1706	70.6014	1680.985714
PC12	1.395	2533.1707	75.6015	1680.033333
MDA-MB231	1.399	2539.8908	82.3216	1680.032653
MCF-7	1.401	2543.2109	85.6417	1679.24902
White matter	1.4121	2561.7712	104.202	1677.971014
Low grade glioma	1.432	2594.8519	137.2827	1674.179268
Glioblastoma	1.447	2619.6124	162.0432	1670.548454

**Table 16:** Performance evaluation table showing position of defect mode, shift in the position of defect mode due to change in the refractive index of sample containing various cancerous cells with respect to the sample containing normal cell and the sensitivity of the proposed structure loaded with different cells under normal incidence *θ*_0_ = 65°.

Cell	Refractive index	Defect mode positions (nm)	Wavelength shift (nm)	Sensitivity (nm/RIU)
Normal	1.35	2404.0081	–	–
Jurkat	1.39	2473.2095	69.2014	1730.035
Hela	1.392	2476.6495	72.6414	1729.557143
PC12	1.395	2481.8496	77.8415	1729.811111
MDA-MB231	1.399	2488.7698	84.7617	1729.830612
MCF-7	1.401	2492.2098	88.2017	1729.445098
White matter	1.4121	2511.3702	107.3621	1728.858293
Low grade glioma	1.432	2545.5709	141.5628	1726.37561
Glioblastoma	1.447	2571.1714	167.1633	1723.33299

**Table 17:** Performance evaluation table showing position of defect mode, shift in the position of defect mode due to change in the refractive index of sample containing various cancerous cells with respect to the sample containing normal cell and the sensitivity of the proposed structure loaded with different cells under normal incidence *θ*_0_ = 70°.

Cell	Refractive index	Defect mode positions (nm)	Wavelength shift (nm)	Sensitivity (nm/RIU)
Normal	1.35	2356.5671	–	–
Jurkat	1.39	2427.3285	70.7614	1769.035
Hela	1.392	2430.8486	74.2815	1768.607143
PC12	1.395	2436.1687	79.6016	1768.924444
MDA-MB231	1.399	2443.2889	86.7218	1769.832653
MCF-7	1.401	2446.8089	90.2418	1769.447059
White matter	1.4121	2466.4893	109.9222	1770.083736
Low grade glioma	1.432	2501.65	145.0829	1769.303659
Glioblastoma	1.447	2528.0506	171.4835	1767.871134

**Table 18:** Performance evaluation table showing position of defect mode, shift in the position of defect mode due to change in the refractive index of sample containing various cancerous cells with respect to the sample containing normal cell and the sensitivity of the proposed structure loaded with different cells under normal incidence *θ*_0_ = 75°.

Cell	Refractive index	Defect mode positions (nm)	Wavelength shift (nm)	Sensitivity (nm/RIU)
Normal	1.35	2317.3663	–	–
Jurkat	1.39	2389.2078	71.8415	1796.0375
Hela	1.392	2392.8479	75.4816	1797.180952
PC12	1.395	2398.248	80.8817	1797.371111
MDA-MB231	1.399	2405.4881	88.1218	1798.404082
MCF-7	1.401	2409.0882	91.7219	1798.468627
White matter	1.4121	2429.1686	111.8023	1800.359098
Low grade glioma	1.432	2465.1293	147.763	1801.987805
Glioblastoma	1.447	2492.1298	174.7635	1801.685567

**Table 19:** Performance evaluation table showing position of defect mode, shift in the position of defect mode due to change in the refractive index of sample containing various cancerous cells with respect to the sample containing normal cell and the sensitivity of the proposed structure loaded with different cells under normal incidence *θ*_0_ = 80°.

Cell	Refractive index	Defect mode positions (nm)	Wavelength shift (nm)	Sensitivity (nm/RIU)
Normal	1.35	2288.0858	–	–
Jurkat	1.39	2360.6472	72.5614	1814.035
Hela	1.392	2364.3273	76.2415	1815.27381
PC12	1.395	2369.8074	81.7216	1816.035556
MDA-MB231	1.399	2377.1275	89.0417	1817.177551
MCF-7	1.401	2380.8076	92.7218	1818.07451
White matter	1.4121	2401.168	113.0822	1820.969404
Low grade glioma	1.432	2437.6488	149.563	1823.939024
Glioblastoma	1.447	2465.0893	177.0035	1824.778351

**Table 20:** Performance evaluation table showing position of defect mode, shift in the position of defect mode due to change in the refractive index of sample containing various cancerous cells with respect to the sample containing normal cell and the sensitivity of the proposed structure loaded with different cells under normal incidence *θ*_0_ = 85°.

Cell	Refractive index	Defect mode positions (nm)	Wavelength shift (nm)	Sensitivity (nm/RIU)
Normal	1.35	2270.0054	–	–
Jurkat	1.39	2342.9669	72.9615	1824.0375
Hela	1.392	2346.6469	76.6415	1824.797619
PC12	1.395	2352.167	82.1616	1825.813333
MDA-MB231	1.399	2359.5672	89.5618	1827.791837
MCF-7	1.401	2363.2473	93.2419	1828.272549
White matter	1.4121	2383.8077	113.8023	1832.565217
Low grade glioma	1.432	2420.6084	150.603	1836.621951
Glioblastoma	1.447	2448.329	178.3236	1838.387629

The average sensitivity of all structures loaded independently with the samples containing different cancerous cells as obtained from Tables 3, 4, 5, 6, 7, 8, 9, 10, 11, 12, 13, 14, 15, 16, 17, 18, 19 and 20 are listed in Table 21 below.

**Table 21:** Angle dependent average sensitivity of the proposed design loaded with various samples under investigation.

Incident angle (degree)	Average sensitivity (nm/RIU)	Source table
0	1175.786124	Table 3
5	1179.753948	Table 4
10	1192.91687	Table 5
15	1214.050023	Table 6
20	1243.758699	Table 7
25	1282.142053	Table 8
30	1327.093822	Table 9
35	1378.479976	Table 10
40	1436.074495	Table 11
45	1496.810724	Table 12
50	1560.038781	Table 13
55	1620.974649	Table 14
60	1678.003995	Table 15
65	1728.405732	Table 16
70	1769.138103	Table 17
75	1798.936843	Table 18
80	1818.785401	Table 19
85	1829.785954	Table 20

**Figure 5:**
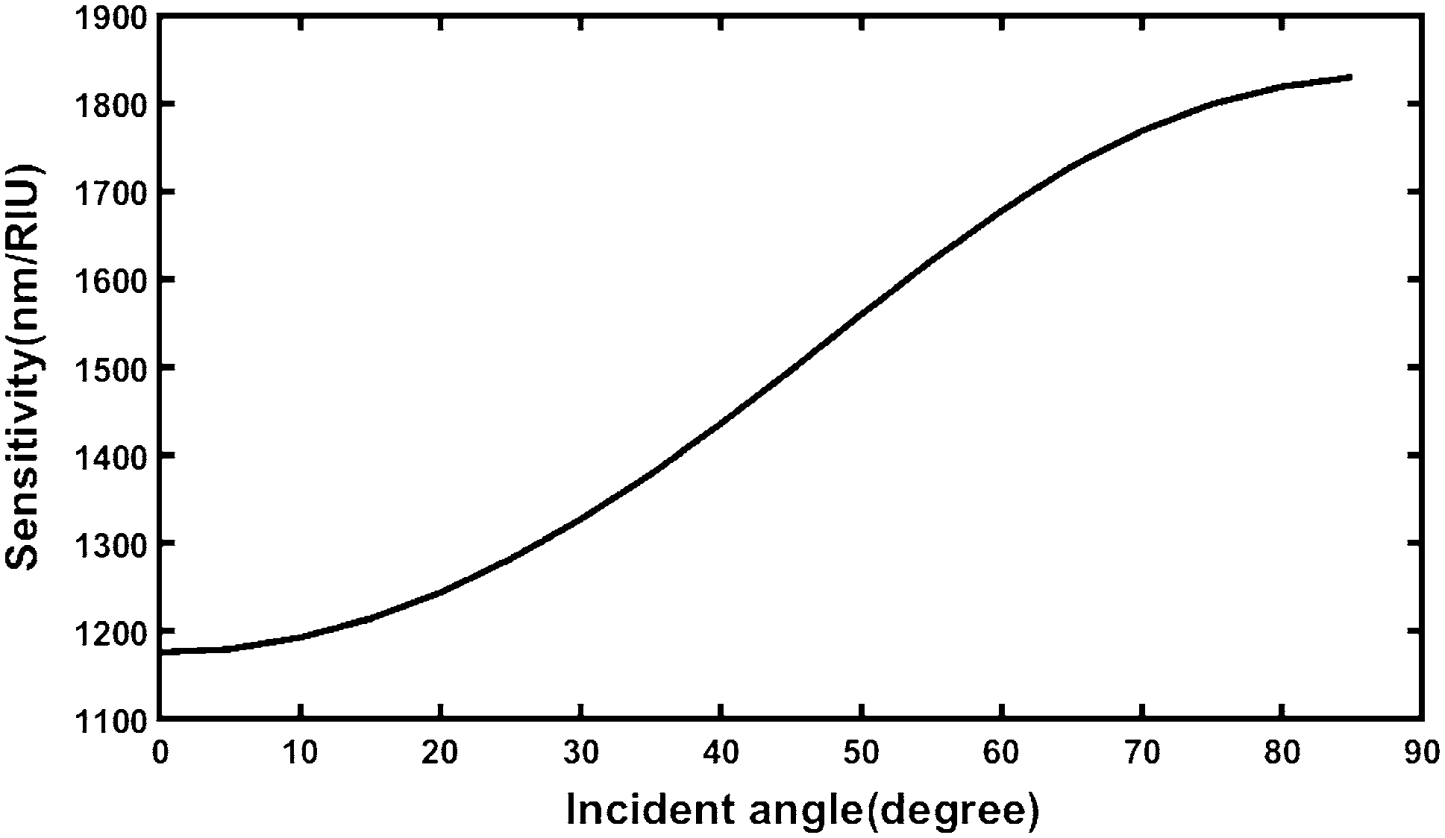
Angle dependent average sensitivity of ternary photonic structure with defect of structural parameters *d*_1_ = 95 nm, *d*_2_ = 155 nm, *d*_3_ = 235 nm, *n*_1_ = 3.3, *n*_2_ = 4.2, *n*_3_ = 1.46, *d*_D_ = 1D and *N* = 5 corresponding to s-polarized wave.

The angle dependent average sensitivity variations of the structure loaded independently with samples are being plotted in Fig. 5 for better understanding. It can be clearly seen from the Fig. 5 that the sensitivity of the proposed designed reaches to maximum when angle of incidence is set to 85°.

We have also investigated the impact of change in the angle of incidence on the full width half maximum of resonant peak which in turn affects the performance of the proposed biosensor composed of 1D ternary photonic structure with defect. For this purpose, we have studied the how the positions of left and right edges of defect mode inside photonic band gap of the structure loaded independently with normal sample of refractive index 1.35 corresponding to θ° = 0°, 5°, 10°, 15°, 20°, 25°, 30°, 35°, 40°, 45°, 50°, 55°, 60°, 65°, 70°, 75°, 80° and 85° under s polarized light vary. In fact, this analysis gives us insight while selecting the bests suitable incident angle for our design. Table 22 summarizes the average shifting of left and right edges of defect mode in PBG of the structure loaded independently with all sample dependent upon the incident angle corresponding to s-polarized light.

**Table 22:** An angle dependent average shifting of left and right edges of defect mode associated with the structure loaded independently with all samples.

Incident angle (degree)	Details of defect mode		
	Left edge (nm)	Right edge (nm)	FWHM (nm)
0	2989.492878	2990.0969	0.6039748
5	2985.698475	2986.2932	0.594675704
10	2974.357527	2974.925	0.567509132
15	2955.598784	2956.1248	0.526037981
20	2929.652758	2930.1256	0.472867167
25	2896.869661	2897.2845	0.414839437
30	2857.749166	2858.1054	0.356274948
35	2812.973423	2813.2742	0.30079363
40	2763.441582	2763.6919	0.25029342
45	2710.299594	2710.5087	0.209076648
50	2654.966803	2655.1411	0.17429161
55	2599.131257	2599.2755	0.144290763
60	2544.714582	2544.84	0.125416417
65	2493.807695	2493.913	0.105310979
70	2448.538265	2448.6312	0.092928298
75	2410.925509	2411.0052	0.079676202
80	2108.822795	2108.8727	0.049866984
85	2365.234679	2365.2917	0.057001243

**Figure 6:**
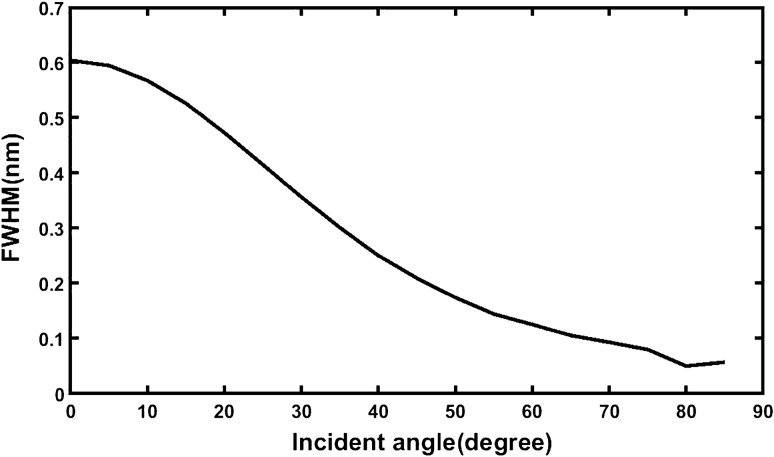
Angle dependent change in the full width half maximum of defect mode associated with ternary photonic structure loaded independently with all samples.

It can be seen from the Table 6 that as incident angle increases from 0° to 85° the left and right band edges of defect mode start moving towards lower wavelength side, also their FWHM decreases. Figure 6 below visualize the data presented in above table. It is evident from the Fig. 6 that as incident angle increases from 0° to 85° the FWHM of defect mode starts reducing and becomes lowest at *θ*_0_ = 80°. Further increase in the incident angle results very minute change in the FWHM of the defect mode at *θ*_0_ = 85° it becomes 0.057001243 nm. Thus *θ*_0_ = 85° has been considered as an optimum value of an incident angle corresponding to which our structure becomes sensitive as evident from Table 20.

### The effect of the cavity layer thickness on the performance at *θ*_0_ = 0°

Next, efforts have further extended to improve the sensitivity of the design. For this purpose, we have fixed incident angle *θ*_0_ = 0° and the samples are being loaded independently into the different structures of cavity thickness *d*_D_ = 1600 nm, 2400 nm, 3200 nm, 4000 nm, 4800 nm, 5600 nm, 6400 nm, 7200 nm and 8000 nm. The defect mode positions of each structure inside PBG loaded independently with all samples along with the shift in the defect mode positions of the structure with respect to normal sample and the respective sensitivity of the structures with cavity thickness *d*_D_ = 1D, 2D, 3D, 4D, 5D, 6D, 7D, 8D, and 9D are being summarized in Tables 23, 24, 25, 26, 27, 28, 29, 30 and 31 respectively.

**Table 23:** Performance evaluation table showing position of defect mode, shift in the position of defect mode due to change in the refractive index of sample containing various cancerous cells with respect to the sample containing normal cell and the sensitivity of the proposed structure loaded independently with sample containing different cells under normal incidence with cavity thickness *d*_D_ = 2D = 1600 nm.

Cell	Refractive index	Defect mode positions (nm)	Wavelength shift (nm)	Sensitivity (nm/RIU)
Normal	1.35	2646.5729	–	–
Jurkat	1.39	2699.374	52.8011	1320.0275
Hela	1.392	2702.014	55.4411	1320.02619
PC12	1.395	2706.0141	59.4412	1320.91556
MDA-MB231	1.399	2711.3342	64.7613	1321.65918
MCF-7	1.401	2713.9743	67.4014	1321.59608
White matter	1.4121	2728.6946	82.1217	1322.41063
Low grade glioma	1.432	2755.1751	108.6022	1324.41707
Glioblastoma	1.447	2775.0955	128.5226	1324.97526

**Table 24:** Performance evaluation table showing position of defect mode, shift in the position of defect mode due to change in the refractive index of sample containing various cancerous cells with respect to the sample containing normal cell and the sensitivity of the proposed structure loaded independently with sample containing different cells under normal incidence *θ*_0_ = 0° with cavity thickness *d*_D_ = 3D = 2400 nm.

Cell	Refractive index	Defect mode positions (nm)	Wavelength shift (nm)	Sensitivity (nm/RIU)
Normal	1.35	2520.2904	–	–
Jurkat	1.39	2574.2515	53.9611	1349.0275
Hela	1.392	2576.9715	56.6811	1349.55
PC12	1.395	2581.0916	60.8012	1351.13778
MDA-MB231	1.399	2586.5317	66.2413	1351.86327
MCF-7	1.401	2589.2518	68.9614	1352.18431
White matter	1.4121	2604.4521	84.1617	1355.26087
Low grade glioma	1.432	2631.8126	111.5222	1360.02683
Glioblastoma	1.447	2652.5331	132.2427	1363.3268

**Table 25:** Performance evaluation table showing position of defect mode, shift in the position of defect mode due to change in the refractive index of sample containing various cancerous cells with respect to the sample containing normal cell and the sensitivity of the proposed structure loaded independently with sample containing different cells under normal incidence *θ*_0_ = 0° with cavity thickness *d*_D_ = 4D = 3200 nm.

Cell	Refractive index	Defect mode positions (nm)	Wavelength shift (nm)	Sensitivity (nm/RIU)
Normal	1.35	2448.409	–	–
Jurkat	1.39	2502.8901	54.4811	1362.0275
Hela	1.392	2505.6501	57.2411	1362.88333
PC12	1.395	2509.8102	61.4012	1364.47111
MDA-MB231	1.399	2515.3303	66.9213	1365.74082
MCF-7	1.401	2518.1304	69.7214	1367.08628
White matter	1.4121	2533.5307	85.1217	1370.71981
Low grade glioma	1.432	2561.3712	112.9622	1377.58781
Glioblastoma	1.447	2582.4516	134.0426	1381.88247

**Table 26:** Performance evaluation table showing position of defect mode, shift in the position of defect mode due to change in the refractive index of sample containing various cancerous cells with respect to the sample containing normal cell and the sensitivity of the proposed structure loaded independently with sample containing different cells under normal incidence *θ*_0_ = 0° with cavity thickness *d*_D_ = 5D = 4000 nm.

Cell	Refractive index	Defect mode positions (nm)	Wavelength shift (nm)	Sensitivity (nm/RIU)
Normal	1.35	2401.608	–	–
Jurkat	1.39	2456.5291	54.9211	1373.0275
Hela	1.392	2459.3292	57.7212	1374.31429
PC12	1.395	2463.4893	61.8813	1375.14
MDA-MB231	1.399	2469.0894	67.4814	1377.17143
MCF-7	1.401	2471.8894	70.2814	1378.06667
White matter	1.4121	2487.4497	85.8417	1382.31401
Low grade glioma	1.432	2515.6103	114.0023	1390.27195
Glioblastoma	1.447	2536.9707	135.3627	1395.49175

**Table 27:** Performance evaluation table showing position of defect mode, shift in the position of defect mode due to change in the refractive index of sample containing various cancerous cells with respect to the sample containing normal cell and the sensitivity of the proposed structure loaded independently with sample containing different cells under normal incidence *θ*_0_ = 0° with cavity thickness *d*_D_ = 6D = 4800 nm.

Cell	Refractive index	Defect mode positions (nm)	Wavelength shift (nm)	Sensitivity (nm/RIU)
Normal	1.35	2368.5674	–	–
Jurkat	1.39	2423.8485	55.2811	1382.0275
Hela	1.392	2426.6485	58.0811	1382.88333
PC12	1.395	2430.8486	62.2812	1384.02667
MDA-MB231	1.399	2436.4887	67.9213	1386.14898
MCF-7	1.401	2439.2888	70.7214	1386.69412
White matter	1.4121	2455.0091	86.4417	1391.97585
Low grade glioma	1.432	2483.4097	114.8423	1400.51585
Glioblastoma	1.447	2504.9701	136.4027	1406.2134

**Table 28:** Performance evaluation table showing position of defect mode, shift in the position of defect mode due to change in the refractive index of sample containing various cancerous cells with respect to the sample containing normal cell and the sensitivity of the proposed structure loaded independently with sample containing different cells under normal incidence *θ*_0_ = 0° with cavity thickness *d*_D_ = 7D = 5600 nm.

Cell	Refractive index	Defect mode positions (nm)	Wavelength shift (nm)	Sensitivity (nm/RIU)
Normal	1.35	2343.8869	–	–
Jurkat	1.39	2399.448	55.5611	1389.0275
Hela	1.392	2402.288	58.4011	1390.50238
PC12	1.395	2406.5281	62.6412	1392.02667
MDA-MB231	1.399	2412.2082	68.3213	1394.31225
MCF-7	1.401	2415.0483	71.1614	1395.32157
White matter	1.4121	2430.8486	86.9617	1400.34944
Low grade glioma	1.432	2459.4492	115.5623	1409.29634
Glioblastoma	1.447	2481.2096	137.3227	1415.69794

**Table 29:** Performance evaluation table showing position of defect mode, shift in the position of defect mode due to change in the refractive index of sample containing various cancerous cells with respect to the sample containing normal cell and the sensitivity of the proposed structure loaded independently with sample containing different cells under normal incidence *θ*_0_ = 0° with cavity thickness *d*_D_ = 8D = 6400 nm.

Cell	Refractive index	Defect mode positions (nm)	Wavelength shift (nm)	Sensitivity (nm/RIU)
Normal	1.35	2324.7265	–	–
Jurkat	1.39	2380.5676	55.8411	1396.0275
Hela	1.392	2383.4077	58.6812	1397.17143
PC12	1.395	2387.6478	62.9213	1398.25111
MDA-MB231	1.399	2393.3679	68.6414	1400.8449
MCF-7	1.401	2396.2079	71.4814	1401.59608
White matter	1.4121	2412.1282	87.4017	1407.43478
Low grade glioma	1.432	2440.9288	116.2023	1417.10122
Glioblastoma	1.447	2462.8093	138.0828	1423.53402

**Table 30:** Performance evaluation table showing position of defect mode, shift in the position of defect mode due to change in the refractive index of sample containing various cancerous cells with respect to the sample containing normal cell and the sensitivity of the proposed structure loaded independently with sample containing different cells under normal incidence *θ*_0_ = 0° with cavity thickness *d*_D_ = 9D = 7200 nm.

Cell	Refractive index	Defect mode positions (nm)	Wavelength shift (nm)	Sensitivity (nm/RIU)
Normal	1.35	2309.3262	–	–
Jurkat	1.39	2365.4473	56.1211	1403.0275
Hela	1.392	2368.2874	58.9612	1403.8381
PC12	1.395	2372.5675	63.2413	1405.36222
MDA-MB231	1.399	2378.3276	69.0014	1408.19184
MCF-7	1.401	2381.1676	71.8414	1408.6549
White matter	1.4121	2397.2079	87.8817	1415.16425
Low grade glioma	1.432	2426.1285	116.8023	1424.41829
Glioblastoma	1.447	2448.129	138.8028	1430.9567

**Table 31:** Performance evaluation table showing position of defect mode, shift in the position of defect mode due to change in the refractive index of sample containing various cancerous cells with respect to the sample containing normal cell and the sensitivity of the proposed structure loaded independently with sample containing different cells under normal incidence *θ*_0_ = 0° with cavity thickness *d*_D_ = 10D = 8000 nm.

Cell	Refractive index	Defect mode positions (nm)	Wavelength shift (nm)	Sensitivity (nm/RIU)
Normal	1.35	2296.6859	–	–
Jurkat	1.39	2353.0471	56.3612	1409.03
Hela	1.392	2355.9271	59.2412	1410.50476
PC12	1.395	2360.2472	63.5613	1412.47333
MDA-MB231	1.399	2366.0073	69.3214	1414.72245
MCF-7	1.401	2368.8874	72.2015	1415.71569
White matter	1.4121	2384.9677	88.2818	1421.60709
Low grade glioma	1.432	2414.0483	117.3624	1431.24878
Glioblastoma	1.447	2436.1287	139.4428	1437.55464

It can be observed from Tables 23, 24, 25, 26, 27, 28, 29, 30 and 31 that the sensitivity of the structure loaded independently with carcinogenic samples with respect to normal sample increases as cavity layer thickness of the structure increases under normal incidence. The average sensitivity of different structures of cavity layer thickness *d*_D_ = 800 nm, 1600 nm, 2400 nm, 3200 nm, 4000 nm, 4800 nm, 5600 nm, 6400 nm, 7200 nm and 8000 nm loaded with various samples under investigation at *θ*_0_ = 0° are presented in Table 32 below. The data presented in Table 32 shows as the structure of cavity layer thickness *d*_D_ = 8000 nm possesses maximum average sensitivity of 1419.107092 nm/RIU under normal incidence.

**Figure 7:**
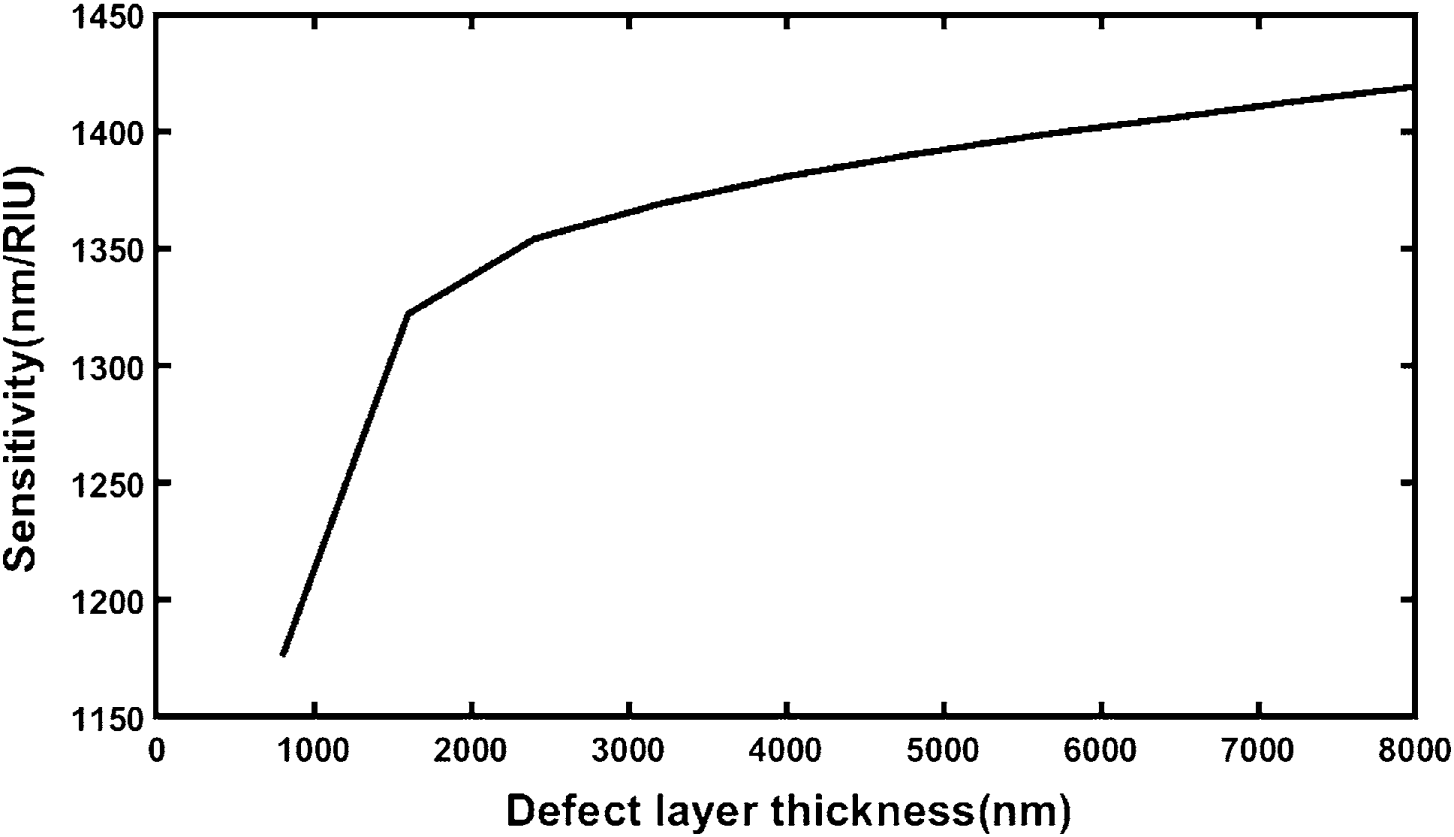
Plot showing average sensitivity of different structures of cavity layer thickness *d*_D_ = 800 nm, 1600 nm, 2400 nm, 3200 nm, 4000 nm, 4800 nm, 5600 nm, 6400 nm, 7200 nm and 8000 nm each loaded with various samples under investigation at *θ*_0_ = 0°.

The pictorial representation of the data of Table 32 has been shown in Fig. 7. It helps us to estimate the optimum value of the cavity layer thickness for the proposed structure. It shows that the average sensitivity of the thickness initially increases rapidly as the cavity layer thickness up to *d*_D_ = 2400 nm. Further increase in the cavity layer thickness results moderate enhancement in the average sensitivity of the structure. After *d*_D_ = 5600 nm the sensitivity increases slowly. The average sensitivity of our design reaches to 1419.107092 nm/RIU which is maximum at *θ*_0_ = 0°.

**Table 32:** Average sensitivity of the proposed design of cavity thickness *d*_D_ = 800 nm, 1600 nm, 2400 nm, 3200 nm, 4000 nm, 4800 nm, 5600 nm, 6400 nm, 7200 nm and 8000 loaded with various samples under investigation at *θ*_0_ = 0°.

Defect layer thickness (nm)	Average sensitivity (nm/RIU)	Source table
1D	1175.786124	Table 22
2D	1322.003433	Table 23
3D	1354.04717	Table 24
4D	1369.04989	Table 25
5D	1380.724699	Table 26
6D	1390.060712	Table 27
7D	1398.31676	Table 28
8D	1405.24513	Table 29
9D	1412.451725	Table 30
10D	1419.107092	Table 31

### The effect of the period number on the performance at *θ*_0_ = 0°

After getting the optimized values of incident angle and cavity layer thickness as 85° and 8000 nm respectively, we have focused our attention to get the optimized value of period number for our design to extract best sensing performance of our design. For this purpose, we have chosen cavity layer thickness *d*_D_ = 800 nm and *θ*_0_ = 0° along with other structural parameters which are identical as discussed above. To get the optimized value of the period number *N* of the structure we have considered four values of *N* = *S* = 3, 4, 5 and 6. The defect mode position inside PBG, shift in the central wavelength and the sensitivity of the structure of cavity thickness *d*_D_ = 800 nm and *θ*_0_ = 0° loaded independently with all samples with respect to normal sample corresponding to *N* = 3, 4 and 6 are being given in Tables 33, 34 and 35 respectively.

**Table 33:** Performance evaluation table showing position of defect mode, shift in the position of defect mode due to change in the refractive index of sample containing various cancerous cells with respect to the sample containing normal cell and the sensitivity of the proposed structure with cavity thickness *d*_D_ = 800 nm and *N* = 3 loaded independently with sample containing different cells under normal incidence *θ*_0_ = 0°.

Cell	Refractive index	Defect mode position (nm)	Wavelength shift (nm)	Sensitivity (nm/RIU)
Normal	1.35	2935.4587	–	–
Jurkat	1.39	2983.5797	48.121	1203.025
Hela	1.392	2985.9797	50.521	1202.88095
PC12	1.395	2989.5398	54.0811	1201.80222
MDA-MB231	1.399	2994.2999	58.8412	1200.84082
MCF-7	1.401	2996.6999	61.2412	1200.80784
White matter	1.4121	3009.8602	74.4015	1198.09179
Low grade glioma	1.432	3033.6207	98.162	1197.09756
Glioblastoma	1.447	3050.781	115.3223	1188.88969

**Table 34:** Performance evaluation table showing position of defect mode, shift in the position of defect mode due to change in the refractive index of sample containing various cancerous cells with respect to the sample containing normal cell and the sensitivity of the proposed structure with cavity thickness *d*_D_ = 800 nm and *N* = 4 loaded independently with sample containing different cells under normal incidence *θ*_0_ = 0°.

Cell	Refractive index	Defect mode position (nm)	Wavelength shift (nm)	Sensitivity (nm/RIU)
Normal	1.35	2929.9386	–	–
Jurkat	1.39	2977.3795	47.4409	1186.0225
Hela	1.392	2979.7396	49.801	1185.7381
PC12	1.395	2983.2597	53.3211	1184.91333
MDA-MB231	1.399	2987.8998	57.9612	1182.88163
MCF-7	1.401	2990.2598	60.3212	1182.76863
White matter	1.4121	3003.2201	73.2815	1180.05636
Low grade glioma	1.432	3026.2205	96.2819	1174.16951
Glioblastoma	1.447	3050.781	115.3223	1188.88969

**Table 35:** Performance evaluation table showing position of defect mode, shift in the position of defect mode due to change in the refractive index of sample containing various cancerous cells with respect to the sample containing normal cell and the sensitivity of the proposed structure with cavity thickness *d*_D_ = 800 nm and *N* = 6 loaded independently with sample containing different cells under normal incidence *θ*_0_ = 0°.

Cell	Refractive index	Defect mode position (nm)	Wavelength shift (nm)	Sensitivity (nm/RIU)
Normal	1.35	2928.4986	–	–
Jurkat	1.39	2975.6595	47.1609	1179.0225
Hela	1.392	2978.0196	49.521	1179.07143
PC12	1.395	2981.4996	53.001	1177.8
MDA-MB231	1.399	2986.1797	57.6811	1177.16531
MCF-7	1.401	2988.4998	60.0012	1176.49412
White matter	1.4121	3001.34	72.8414	1172.9694
Low grade glioma	1.432	3024.1805	95.6819	1166.85244
Glioblastoma	1.447	3041.2208	112.7222	1162.08454

**Figure 8:**
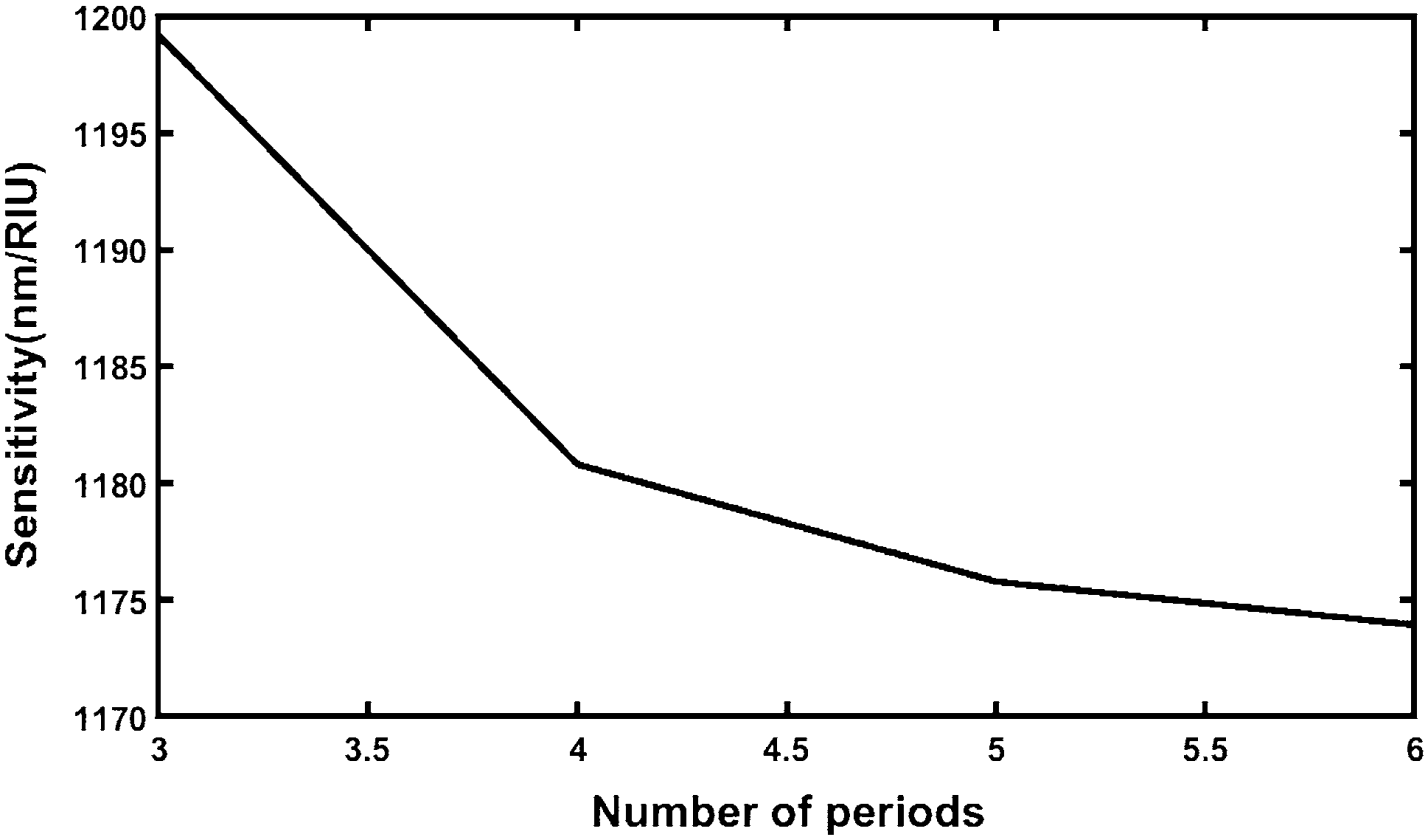
Plot showing average sensitivity of different structures of period number *N* = 3, 4, 5 and 6 with cavity layer thickness *d*_D_ = 800 nm under normal incidence.

The average sensitivity of the structures of cavity thickness *d*_D_ = 800 nm at *θ*_0_ = 0° corresponding to period number *N* = 3, 4, 5 and 6 from the data presented in Tables 33, 34 and 35 respectively are being shown in Table 36. The pictorial representation of the data presented in Table 36 is shown in Fig. 8.

**Table 36:** Average sensitivity of the proposed design of cavity thickness *d*_D_ = 800 nm loaded with various samples under investigation at *θ*_0_ = 0° corresponding to period number *N* = 3, 4, 5 and 6.

Number of periods (*N*)	Average sensitivity (nm/RIU)	Source table
3	1199.179484	Table 33
4	1180.808835	Table 34
5	1175.786124	Table 3
6	1173.932466	Table 35

It is evident form Fig. 8 that the increase in the period number results the drastic fall in the sensitivity of the structure which reaches to minimum when the period number *N* goes to 6. Thus *N* = 3 is the optimized value for our design which corresponds to maximum average sensitivity of 1199.179484 nm/RIU under normal incidence with *d*_D_ = 800 nm.

Additionally, we have also examined the impact of period number on the average shifting of left and right edges of defect mode for determination the FWHM of our design loaded with all samples independently under normal incidence with cavity of thickness *d*_D_ = 800 nm. The shift in the left and right edges of defect mode due to change in the period number of the structure is presented in Table 37 below.

**Table 37:** Period number dependent average shifting of left and right edges of defect mode associated with the structure loaded with all samples independently at *θ*_0_ = 0° and *d*_D_ = 800 nm.

Number of period (*N*)	Details of defect mode		
	Left edge (nm)	Right edge (nm)	FWHM (nm)
3	2991.9627	3003.53598	11.5732787
4	2989.96273	2992.574252	2.61152708
5	2989.49288	2990.096852	0.6039748
6	2651.46676	2651.603966	0.13720366

**Figure 9:**
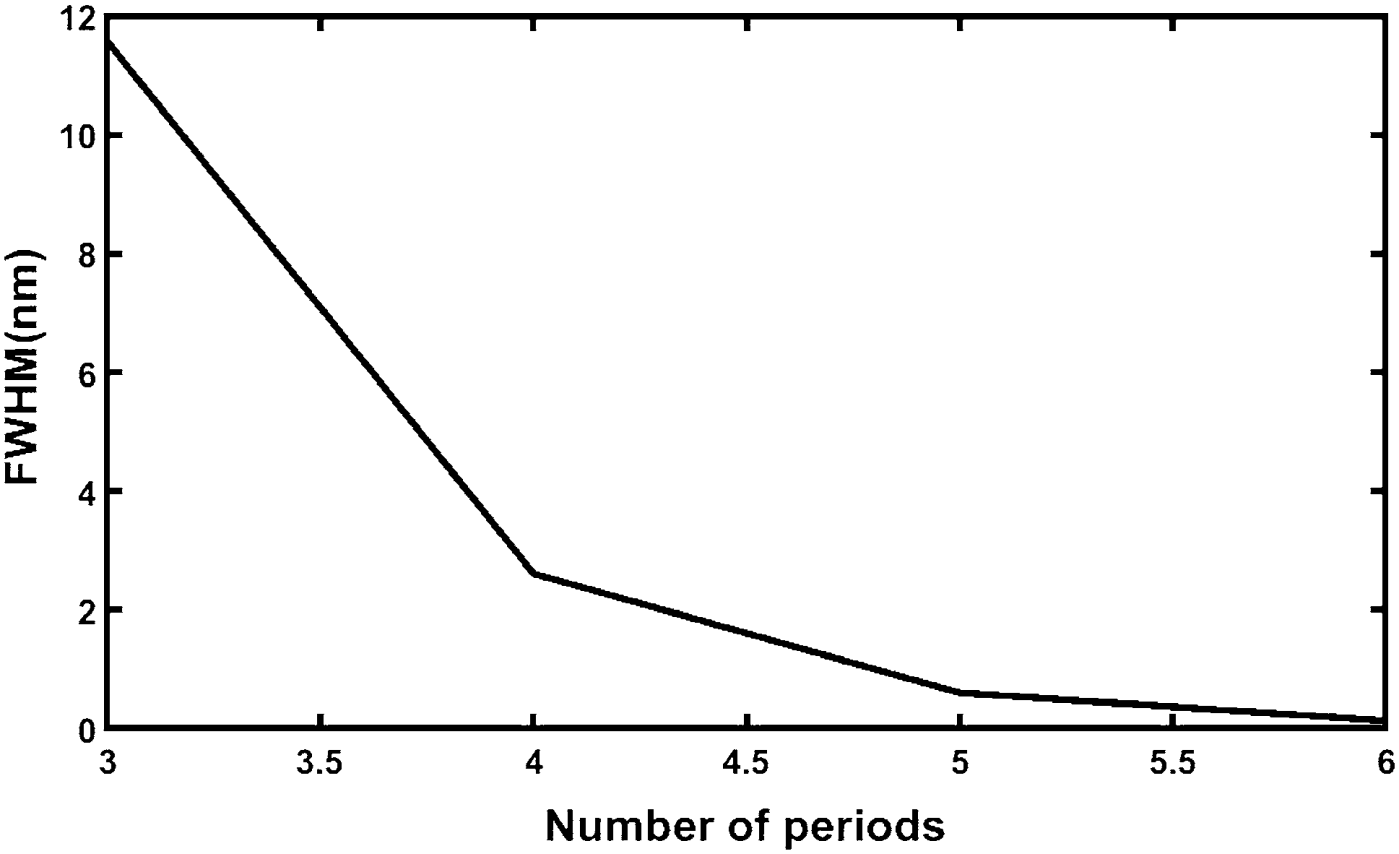
Depiction of period number dependent average shifting of left and right edges of defect mode associated with the structure loaded with all samples independently at *θ*_0_ = 0° and *d*_D_ = 800 nm.

The pictorial representation of the data has been shown in the Fig. 9 below. It shows that the average value of the FWHM of the defect mode corresponding to optimum period number 3 is 11.5732787 nm. Any deviation in the period number from 3 results the deduction in the FWHM value of our design which reaches to minimum at *N* = 6.

### Analysis of the performance of the structure with optimized parameters

**Figure 10:**
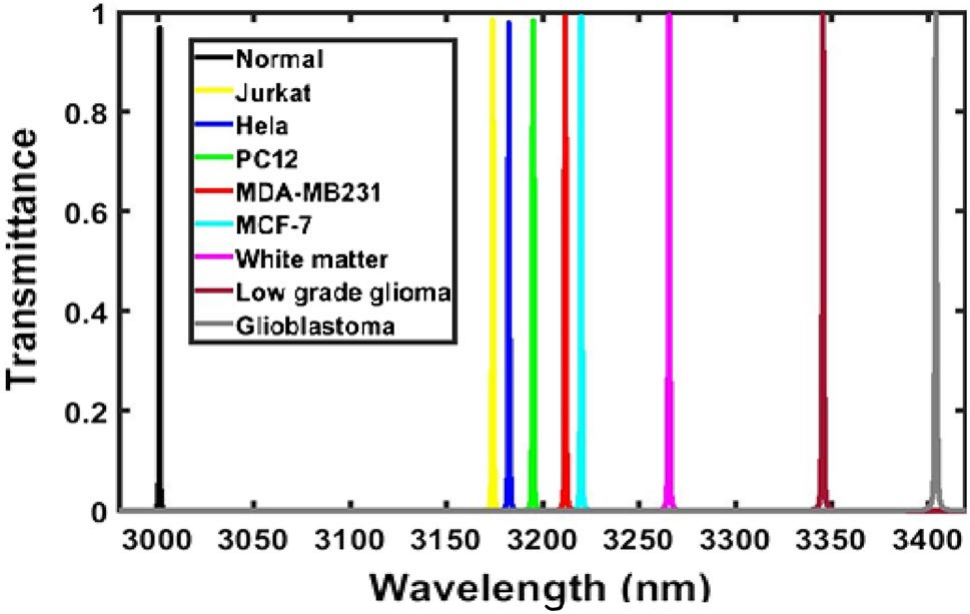
Transmission spectra of the proposed design {air/(Si/Pbs/SiO_2_)^*N*^/Defect /(Si/Pbs/SiO_2_)^S^/air} loaded with samples containing normal, Jurkat, Hela, PC12, MDA-MB231, MCF-7, White matter, Low grade glioma and Glioblastoma cells independently at *θ*_0_ = 85°, *d*_1_ = 95 nm, *d*_2_ = 155 nm, *d*_3_ = 235 nm, *d*_D_ = 8000 nm, *n*_1_ = 3.3, *n*_2_ = 4.2, *n*_3_ = 1.46 and *N* = 3. The black, yellow, blue, green, red, cyan, pink, brown and silver colour defect modes are corresponding to the sample containing normal, Jurkat, Hela, PC12, MDA-MB231, MCF-7, White matter, Low grade glioma and Glioblastoma cells respectively.

After optimizing the incident angle corresponding to s-polarized incident wave, thickness of the cavity region and period number of the structure as 85°, 8000 nm and 3 respectively, we have given our efforts to analyze the performance of the under the influence of optimized external and internal parameters of the proposed structure as *θ*_0_ = 85° and *d*_D_ = 8000 nm and *N* = 3 respectively. First, we have designed the defective ternary photonic structure of period number 3 and cavity thickness 8000 nm which is loaded independently with all samples. The s-polarized incident light is allowed pass through the structure at an incident angle 85°. The transmitted energy of defect mode located inside PBG of the structure is measured with the help of an optical spectrum analyzer via output port. The transmittance spectra of the proposed defective ternary photonic structure {air/(Si/Pbs/SiO_2_)^*N*^/Defect/(Si/Pbs/SiO_2_)^S^/air} loaded independently with sample containing normal cells as well as 8 carcinogenic cells at *θ*_0_ = 85° with *d*_1_ = 95 nm, *d*_2_ = 155 nm, *d*_3_ = 235 nm, *d*_*D*_ = 8000 nm, *n*_1_ = 3.3, *n*_2_ = 4.2, *n*_3_ = 1.46 and *N* = 3 are being shown in Fig. 10. It can be clearly seen from Fig. 10 that as sample containing various cells is changed from normal to Glioblastoma in accordance with Table 2 the position of defect mode inside PBG starts moving towards higher wavelength side due to increase in the refractive indices of the samples. Moreover, FWHM of the defect mode also increases due to this movement which becomes maximum corresponding to sample containing Glioblastoma cells. This increase in the FWHM of the defect mode does not have any impact on the resolution of the peak.

The defect mode position inside PBG, shift in the central wavelength of defect mode and the sensitivity of the structure of cavity thickness *d*_D_ = 8000 nm and period number *N* = 3 loaded independently with all samples with respect to normal sample at *θ*_0_ = 85° corresponding to s-polarized wave have been obtained with the help of Fig. 10. These values have been recorded in Table 38 below. It is evident from the data of Table 38 that as the refractive index of the sample under investigation increases the defect mode position shifted to higher wavelength side in such a way that the shift in the defect mode position associated with sample also increases with respect to the position of defect mode due to sample containing normal cells. Additionally, the sensitivity of the structure under optimized condition varies from maximum of 4325.0875 nm/RIU to minimum of 4155.13505 nm/RIU corresponding to samples containing Jurkat to Glioblastoma cells respectively. Under optimum conditions the average sensitivity of the proposed structure becomes 4270.525928 nm/RIU which is tremendously enhanced.

**Table 38:** Performance evaluation table showing position of defect mode, shift in the position of defect mode due to change in the refractive index of sample containing various cancerous cells with respect to the sample containing normal cell and the sensitivity of the proposed structure with optimized values of cavity thickness *d*_D_ = 8000 nm and period number *N* = 3 loaded independently with sample containing different cells at *θ*_0_ = 85° corresponding to s polarized incident light.

Cell	Refractive index	Defect mode positions (nm)	Wavelength shift (nm)	Sensitivity (nm/RIU)
Normal	1.35	3001.1	–	–
Jurkat	1.39	3174.1035	173.0035	4325.0875
Hela	1.392	3182.5037	181.4037	4319.13571
PC12	1.395	3195.1039	194.0039	4311.19778
MDA-MB231	1.399	3211.7442	210.6442	4298.86122
MCF-7	1.401	3220.0644	218.9644	4293.41961
White matter	1.4121	3265.6653	264.5653	4260.31079
Low grade glioma	1.432	3345.5869	344.4869	4201.05976
Glioblastoma	1.447	3404.1481	403.0481	4155.13505

### Analysis of the optimized structure based on other important sensing parameters

This section deals with the analysis of the proposed biosensing structure composed of one-dimensional ternary photonic structure by means of some other important sensing parameters which are very popular and significant in the design and development of plasmonic and photonic biosensors. In addition to sensitivity we have extended the performance evaluation of the proposed structure by calculating numeric values of quality factor (QF), detection limit (DL), sensor resolution (SR), signal-to-noise ratio (SNR), detection range (DR), detection accuracy (DA), figure of merit (FOM) and standard deviation ${(\sigma }_{peak})$ of our optimized design loaded independently with sample containing normal and eight carcinogenic cells separately^32–42^. All these calculated values have been listed in Table 39 below.

**Table 39:** Performance evaluation table showing numeric values of quality factor (QF), detection limit (DL), sensor resolution (SR), signal-to-noise ratio (SNR), detection range (DR), detection accuracy (DA), figure of merit (FOM) and standard deviation ${(\sigma }_{peak})$ due to change in the refractive index of sample containing various cancerous cells with respect to the sample containing normal cell of our optimized structure with cavity thickness *d*_D_ = 8000 nm and period number *N* = 3 at *θ*_0_ = 85° corresponding to s-polarized incident light.

Cell	Performance evaluating parameters							
	QF	DL*10^–6^	SR	SNR	DR	DA	FOM	σ_peak_
Normal	15,037.89162	–	–	–	6717.902689	5.0107932	–	–
Jurkat	10,796.47238	9.20077	0.03979413	588.4582875	5853.983326	3.4014242	14,711.45719	0.01326471
Hela	10,480.94351	9.48006	0.04094566	597.4170375	5775.434313	3.2933013	14,224.21518	0.01364855
PC12	10,206.50294	9.70215	0.04182791	619.7298863	5710.589931	3.1944197	13,771.77525	0.01394264
MDA-MB231	9903.752039	9.96195	0.04282505	649.543611	5639.886361	3.0836055	13,255.99206	0.01427502
MCF-7	9705.323927	1.01644	0.04363988	659.9620897	5590.328082	3.0140155	12,940.43313	0.01454663
White matter	8665.496908	0.114566	0.04880882	702.0284011	5319.644026	2.6535165	11,304.80517	0.01626961
Low grade glioma	6946.162565	0.147797	0.06209045	715.2293695	4820.683612	2.0762165	8722.309384	0.02069682
Glioblastoma	5842.410243	0.182287	0.07574257	691.7361638	4459.644574	1.7162621	7131.300658	0.02524752

It has been observed from the data of Table 39 that a FOM value of the proposed sensor varies from 14,711.45719 to 7131.300658 which is high as expected. Additionally, QF and DR values of the proposed structure are also high. The order of the detection limit of the structure is quite low and highlights the minute detection capabilities of our design. The SR, SNR, DA and ${\sigma }_{peak}$ values obtained from transmission spectra as shown in Fig. 10 are as per our expectation. These values are supporting our claim of designing ultra-high sensitive biosensor for detection of carcinogenic cells with respect to normal cell.

### Comparing between the sensitivity of the current work with earlier reported work

Finally, we have compared our findings with the recent work published by various research groups between 2018 and 2021. In this comparison authors have surveyed various biosensing structures based on photonic refractometric technology. The classified information based on the survey has been presented in Table 40 below. It shows that the proposed structure can be used for designing of ultra-high sensitive biophotonic sensors. This study may provide technological designing insight to the people who are working in the fabrication of biophotonic sensors.

**Table 40:** Comparison between the sensitivity of proposed photonic structure with earlier work.

Techniques/structures	Year	Sensitivity (nm/RIU)	References
Porous silicon-based Bragg-grating resonator for biosensing	2018	387	^36^
A square lattice of rods in SiO_2_	2018	720	^37^
1D nano composite material coated PC	2019	43	^38^
1D-PC that contains a defect layer	2019	2200	^39^
An array of split-ring resonators detector	2019	658	^40^
Square lattice defect-based PC waveguide sensor	2020	2360.12	^41^
1D-binary PC with additional layers on sides of the sample layer	2021	161	^42^
1D-ternary PC that contains the cancer cell as a defect layer	2021	3282.09	^24^
Current work	2023	4270.525928	–

## Conclusion

In conclusion, we have theoretically examined the biosensing capabilities of 1D defective ternary photonic crystal for accurate and minute identification of cancer cells with respect to normal cell. Both MATLAB computational software and transfer matrix method have been used for obtaining result of this manuscript. The present biosensor works on the principal of refractometric sensing. We have demonstrated how the change in the period number, incident angle corresponding to s-polarized light and thickness of defect layer can be studied to obtain optimized values of these parameters under which our design possesses ultra-high sensitivity. After obtaining optimized values of theses parameters we have calculated QF, DL, SR, SNR, DR, DA, FOM and ${\sigma }_{peak}$ values of our structure for analyzing its overall performance. The average sensitivity of 4270.525928 nm/RIU can be achieved from our design. FOM, QF and DR values of proposed structure are high whereas DL is low of order 10^–6^. The idea of this manuscript may be very helpful for designing of photonic and plasmonic biosensors.

## Data Availability

The data that support the findings of this study are available from the corresponding author upon reason-able request.
